# ﻿Morphological and phylogenetic characterisation of two new soil-borne fungal taxa belonging to Clavicipitaceae (Hypocreales, Ascomycota)

**DOI:** 10.3897/mycokeys.98.106240

**Published:** 2023-06-07

**Authors:** Zhi-Yuan Zhang, Yao Feng, Shuo-Qiu Tong, Chen-Yu Ding, Gang Tao, Yan-Feng Han

**Affiliations:** 1 College of Eco-Environmental Engineering, Guizhou Minzu University, Guiyang 550025, China Guizhou Minzu University Guiyang China; 2 School of Chinese Ethnic Medicine, Guizhou Minzu University, Guiyang, Guizhou, 550025, China Guizhou University Guiyang China; 3 College of Life Sciences, Guizhou University, Guiyang 550025, China Guizhou Minzu University Guiyang China; 4 Institute of Fungus Resources, College of Life Sciences, Guizhou University, Guiyang 550025, China Guizhou University Guiyang China

**Keywords:** Clavicipitaceae, entomopathogenic fungi, new taxa, phylogeny, *Pochonia*, taxonomy

## Abstract

The fungal taxa belonging to the Clavicipitaceae (Hypocreales, Ascomycota) are widely distributed and include diverse saprophytic, symbiotic and pathogenic species that are associated with soils, insects, plants, fungi and invertebrates. In this study, we identified two new fungal taxa belonging to the family Clavicipitaceae that were isolated from soils collected in China. Morphological characterisation and phylogenetic analyses showed that the two species belong to *Pochonia* (*Pochoniasinensis***sp. nov.**) and a new genus for which we propose *Paraneoaraneomyces***gen. nov.** in Clavicipitaceae.

## ﻿Introduction

Fungi are found in a wide array of ecological niches and play key roles as decomposers, mutualists and pathogens (Araújo et al. 2022). Clavicipitaceae (Ascomycota, Hypocreales) is a large fungal family with diverse ecological characteristics and includes saprophytes, symbionts and pathogens that are associated with soils, insects, plants, fungi and other invertebrates (Gams and Zare 2003; Spatafora et al. 2007; Sung et al. 2007a; Steiner et al. 2011; Kepler et al. 2012a). Currently, the family Clavicipitaceae includes 52 genera and more than 500 species (Hyde et al. 2020; Mongkolsamrit et al. 2020a, 2021; Gao et al. 2021; Chen et al. 2022). Some members of these genera are valuable as biocontrol agents in agriculture and production of antibiotics in the pharmaceutical industry (e.g. cyclosporin, fingolimod, hydroxyfungerins; Uchida et al. (2005); Mapook et al. (2022)). For example, species of *Metarhizium* are commercially used as biocontrol agents (Kim et al. 2020). Gao et al. (2021) reported two new entomopathogenic species belonging to the genus *Parametarhizium* (*P.hingganense* and *P.changbaiense*) that were isolated from the forest litters in northeast China and exhibited anti-insect activities against three farmland pests (*Monoleptahieroglyphica*, *Callosobruchuschinensis* and *Rhopalosiphummaidis*).

Phylogenetic analyses showed that the VerticilliumsectionProstrata was heterogenous and *Pochonia* was recognised as a distinct genus with several species that often form dictyochlamydospores and were parasitic on the nematode cysts and eggs (Zare et al. 2001). *Pochoniachlamydosporia* was the first recognised species of the genus *Pochonia*. Subsequently, several new taxa have been identified in this genus. Kepler et al. (2012b, 2014) showed that the genus *Pochonia* belonged to Claviciptaceae; *Pochonia* was polyphyletic and formed two different clades; *P.chlamydosporia* was the only species assigned to the monophyletic clade in the *Pochonia* genus, whereas the remaining species were transferred to a new genus, *Metapochonia*. Currently, *Pochonia* includes three species (*P.globispora*, *P.boninensis* and *P.chlamydosporia*) and four varieties (P.chlamydosporiavar.ellipsospora, P.chlamydosporiavar.catenulata, P.chlamydosporiavar.spinulospora and P.chlamydosporiavar.chlamydosporia). The species of *Pochonia* are commonly obtained from soil and demonstrate the ability to parasitise plant-parasitic nematodes (Nonaka et al. 2013).

In this study, we report the morphological and phylogenetic characterisation of two new taxa belonging to the family Claviciptaceae that were isolated from the urban soil samples in China.

## ﻿Materials and methods

### ﻿Fungal isolation and morphology

The soil samples were collected in June 2020 from the Cengong County (27°16’98’’N, 108°81’46’’E) in Kaili City, Guizhou Province, China. The fungi were isolated using the methods described previously (Zhang et al. 2023). Colonies on PDA were incubated after 14 days at 25 °C and the cultures were placed to slowly dry at 50 °C to produce the holotypes, which were deposited in the Institute of Fungus Resources, Guizhou University, Guiyang City, Guizhou, China (GZUIFR). All living cultures were stored in a metabolically inactive state (i.e. kept in sterile 30% glycerol in a –80 °C freezer) and were deposited in the GZUIFR.

The phenotype was determined by growing the single isolates in plates containing potato dextrose agar (PDA), malt extract agar (MEA), oatmeal agar (OA) and synthetic low-nutrient agar (SNA) medium. The plates were incubated in the dark at 25 °C for 14 days. The photomicrographs of the fungal structures were obtained using an OLYMPUS BX53 microscope equipped with differential interference contrast (DIC) optics, an OLYMPUS DP73 high-definition colour camera and the cellSens software version 1.18.

### ﻿DNA extraction, PCR amplification and sequencing

Total DNA was extracted using the 5% chelex-100 solution as described previously (Zhang et al. 2023). The small subunit (SSU) rDNA, the internal transcribed spacer (ITS), the large subunit (LSU) rDNA, the second largest subunit of RNA polymerase II (*RPB2*) and the translation elongation factor EF-1α (*EF1A*) were PCR amplified and sequenced using primers listed in Table 1. The novel sequences identified in this study were deposited in the GenBank database (Table 2).

**Table 1.** Sequences of primers used in this study.

**Molecular marker**	**Primer name**	**Primer sequence (5´-3´)**	**Reference**
SSU	NS1	GTAGTCATATGCTTGTCTC	White et al. (1990)
	NS4	CTTCCGTCAATTCCTTTAAG	White et al. (1990)
ITS	ITS1	TCCGTAGGTGAACCTGCG	White et al. (1990)
	ITS4	TCCTCCGCTTATTGATATGC	White et al. (1990)
LSU	LR0R	ACCCGCTGAACTTAAGC	Moncalvo et al. (2000)
	LR7	TACTACCACCAAGATCT	Vilgalys and Hester (1990)
*EF1A*	2218R	ATGACACCRACRGCRACRGTYTG	Rehner and Buckley (2005)
	983F	GCYCCYGGHCAYCGTGAYTTYAT	Rehner and Buckley (2005)
*RPB2*	fRPB2-5F	GAYGAYMGWGATCAYTTYGG	Liu et al. (1999)
	RPB2-7cR	CCCATRGCTTGYTTRCCCAT	Liu et al. (1999)

**Table 2.** GenBank accession numbers of the sequences used in this study.

Species	Strains	SSU	ITS	LSU	*RPB2*	*EF1A*	References
*Aciculosporiumoplismeni*	MAFF 246966	–	LC571760	LC571760	LC572054	LC572040	Tanaka et al. (2021)
*Aciculosporiumtake*	MAFF 241224	–	LC571753	LC571753	LC572048	LC572034	Tanaka et al. (2021)
	TNS-F-60465	–	LC571755	LC571756	LC572049	LC572035	Tanaka et al. (2021)
*Aschersoniaconfluens*	BCC 7961	–	JN049841	DQ384947	DQ452465	DQ384976	Kepler et al. (2012b)
*Aschersoniaplacenta*	BCC 7869	EF469121	JN049842	EF469074	EF469104	EF469056	Sung et al. (2007ab); Kepler et al. (2012b)
*Atkinsonellahypoxylon*	B4728	–	–	–	KP689514	KP689546	Young et al. (2015)
*Balansiaepichloe*	A.E.G. 96-15a	–	JN049848	–	EF468908	EF468743	Sung et al. (2007a); Kepler et al. (2012b)
*Balansiahenningsiana*	A.E.G. 96-27a	AY545723	JN049815	AY545727	DQ522413	AY489610	Castlebury et al. (2004); Lutzoni et al. (2004); Spatafora et al. (2007); Kepler et al. (2012b)
*Clavicepsfusiformis*	ATCC 26019	DQ522539	JN049817	U17402	–	DQ522320	Rehner et al. (1995); Spatafora et al. (2007); Kepler et al. (2012b)
*Clavicepspurpurea*	GAM 12885	–	U57669	AF543789	DQ522417	AF543778	Currie et al. (2003); Spatafora et al. (2007)
	SA cp 11	EF469122		EF469075	EF469105	EF469058	Sung et al. (2007ab)
*Collarinaaurantiaca*	FMR 11134	–	KJ807178	KJ807181	–	–	Crous et al. (2014)
	FMR 11784	–	KJ807177	KJ807180	–	–	Crous et al. (2014)
*Conoideocrellaluteorostrata*	NHJ 11343	EF468995	JN049859	–	–	EF468801	Sung et al. (2007a); Kepler et al. (2012b)
	NHJ 12516	EF468994	JN049860	–	EF468946	EF468800	Sung et al. (2007a); Kepler et al. (2012b)
*Conoideocrellatenuis*	NHJ 6293	EU369112	JN049862	EU369044	EU369087	EU369029	Johnson et al. (2009); Kepler et al. (2012b)
*Corallocytostromaornithocopreoides*	WAC 8705	–	–	–	LT216620	LT216546	Píchová et al. (2018)
*Dussiellatuberiformis*	J.F.White	–	–	–	JQ257020	JQ257027	Kepler et al. (2012a)
*Ephelisjaponica*	CBS 236.64	–	MH858427	–	–	–	Vu et al. (2019)
	Eph.oryzae	–	AB038564	–	–	–	Tanaka et al. (2001)
*Ephelistripsaci*	CBS 857.72	–	KP859042	KP858978	–	–	Hernández-Restrepo et al. (2016)
*Epichloëelymi*	C.Schardl 760	–	–	AY986924	–	AY986951	Chaverri et al. (2005a)
*Epichloëtyphina*	ATCC 56429	–	JN049832	U17396	DQ522440	AF543777	Rehner et al. (1995); Currie et al. (2003); Spatafora et al. (2007); Kepler et al. (2012b)
*Helicocollumsurathaniense*	BCC 34463	–	–	KT222328	–	KT222336	Luangsa-ard et al. (2017a)
	BCC 34464	–	–	KT222329	–	KT222337	Luangsa-ard et al. (2017a)
*Heteroepichloebambusae*	Ba-01	–	AB065426	–	–	–	Tanaka et al. (2002)
	Bo-01	–	AB065428	–	–	–	Tanaka et al. (2002)
*Heteroepichloesasae*	E.sasae-H	–	AB065432	–	–	–	Tanaka et al. (2002)
	E.sasae-N	–	AB065431	–	–	–	Tanaka et al. (2002)
*Keithomycescarneus*	CBS 239.32	EF468988	NR_131993	NG_057769	EF468938	EF468789	Sung et al. (2007a)
*Keithomyces* sp.	CBS 126563	MT078871	MT078883	MT078856	MT078921	–	Mongkolsamrit et al. (2020a)
*Marquandomycesmarquandii*	CBS 182.27	EF468990	MH854923	MH866418	EF468942	EF468793	Sung et al. (2007a); Vu et al. (2019)
*Marquandomyces* sp.	CBS 127132	MT078872	MT078882	MT078857	MT078922	–	Mongkolsamrit et al. (2020a)
*Metapochoniabulbillosa*	JCM 18596	AB758252	AB709836	AB709809	AB758690	AB758460	Nonaka et al. (2013)
	CBS 145.70	AF339591	MH859529	AF339542	EF468943	EF468796	Sung et al. (2001); Sung et al. (2007a); Vu et al. (2019)
*Metapochoniacordycipiticonsociata*	CGMCC 3.17365	KM263572	KM263569	KM263573	KM263579	KM263584	Huang et al. (2015)
	CGMCC 3.17366	KM263570	KM263567	KM263574	KM263580	KM263582	Huang et al. (2015)
*Metapochoniagoniodes*	CBS 891.72	AF339599	AJ292409	AF339550	DQ522458	DQ522354	Zare et al. (2000); Sung et al. (2001); Spatafora et al. (2007)
*Metapochoniamicrobactrospora*	CBS 101433	–	AJ292408	AF339538	KJ398701	KJ398794	Zare et al. (2000); Kepler et al. (2014)
*Metapochoniarubescens*	CBS 464.88	AF339615	MH862138	MH873830	EF468944	EF468797	Sung et al. (2001); Sung et al. (2007a); Vu et al. (2019)
	JCM 18620	AB758247	AB709859	AB709832	AB758685	AB758455	Nonaka et al. (2013)
Metapochoniasuchlasporiavar.catenata	CBS 248.83	–	MH861579	MH873310	KJ398696	KJ398789	Kepler et al. (2014); Vu et al. (2019)
	CBS 251.83	–	MH861580	MH873311	KJ398697	KJ398790	Kepler et al. (2014); Vu et al. (2019)
*Metarhiziopsismicrospora*	CEHS133a	–	EF464589	EF464571	–	–	Marcelino et al. (2009)
	INEHS133a	–	EF464583	EF464572	–	–	Marcelino et al. (2009)
*Metarhiziumanisopliae*	ARSEF 7487	–	HQ331446	–	DQ468370	DQ463996	Bischoff et al. (2006); Schneider et al. (2011)
	CBS 130.71	MT078868	MT078884	MT078853	MT078918	MT078845	Mongkolsamrit et al. (2020a)
*Metarhiziumflavoviride*	CBS 125.65	MT078869	MT078885	MT078854	MT078919	MT078846	Mongkolsamrit et al. (2020a)
	CBS 700.74	MT078870	–	MT078855	MT078920	MT078847	Mongkolsamrit et al. (2020a)
	CBS 218.56	–	MH857590	MH869139	KJ398694	KJ398787	Kepler et al. (2014); Vu et al. (2019)
*Moelleriellaphyllogena*	CUP 067785	–	–	EU392610	–	EU392674	Chaverri et al. (2008)
	CUP 067793	–	–	EU392608	–	EU392672	Chaverri et al. (2008)
*Moelleriellaumbospora*	CUP 067817	–	–	EU392628	–	EU392688	Chaverri et al. (2008)
*Morakotiafusca*	BCC 64125	–	–	KY794862	–	KY794857	Mongkolsamrit et al. (2021)
	BCC 79272	–	–	KY794861	–	KY794856	Mongkolsamrit et al. (2021)
*Mycophilomycespericoniae*	CPC 27558	–	KY173418	KY173509	–	–	Crous et al. (2013)
*Myriogenosporaatramentosa*	A.E.G 96-32	AY489701	–	AY489733	DQ522455	AY489628	Castlebury et al. (2004); Spatafora et al. (2007)
*Neoaraneomycesaraneicola*	DY101711	–	MW730520	MW730609	MW753026	MW753033	Chen et al. (2022)
	DY101712	–	MW730522	MW730610	MW753027	MW753034	Chen et al. (2022)
*Neobaryaparasitica*	Marsons/n	–	KP899626	KP899626	–	–	Lawrey et al. (2015)
*Niessliaexilis*	CBS 560.74	AY489688	MG827005	AY489720	–	AY489614	Castlebury et al. (2004)
*Nigeliaaurantiaca*	BCC 13019	GU979939	–	GU979948	GU979971	GU979957	Luangsa-ard et al. (2017b)
*Nigeliamartialis*	EFCC 6863	–	–	JF415974	–	JF416016	Kepler et al. (2012b)
*Orbiocrellapetchii*	NHJ 6209	EU369104	JN049861	EU369039	EU369081	EU369023	Johnson et al. (2009); Kepler et al. (2012b)
	NHJ 6240	EU369103	–	EU369038	EU369082	EU369022	Johnson et al. (2009)
*Papiliomycesliangshanensis*	EFCC 1452	EF468962	–	EF468815	–	EF468756	Sung et al. (2007a)
	EFCC 1523	EF468961	–	EF468814	EF468918	EF468755	Sung et al. (2007a)
*Papiliomycesshibinensis*	GZUH SB13050311	KR153588	KR153585	–	–	KR153589	Wen et al. (2015)
*Parametarhiziumchangbaiense*	CGMCC 19143	MN590231	MN589741	MN589994	MT921829	MN908589	Gao et al. (2021)
*Parametarhiziumhingganense*	CGMCC 19144	MN055706	MN055703	MN061635	MT939494	MN065770	Gao et al. (2021)
***Paraneoaraneomycessinensis***	**ZY 22.006**	**OQ709248**	**OQ709254**	**OQ709260**	**OQ719621**	**OQ719626**	**This study**
	**ZY 22.007**	**OQ709249**	**OQ709255**	**OQ709261**	**OQ719622**	**OQ719627**	**This study**
	**ZY 22.008**	**OQ709250**	**OQ709256**	**OQ709262**	**OQ719623**	**OQ719628**	**This study**
*Parepichloecinerea*	Ne-01	–	AB065425	–	–	–	Tanaka et al. (2002)
*Periglandulaipomoeae*	IasaF13	–	–	–	KP689517	KP689568	Steiner et al. (2011)
*Pochoniaboninensis*	JCM 18597	AB758255	AB709858	AB709831	AB758693	AB758463	Nonaka et al. (2013)
*Pochoniachlamydosporia*	CBS 101244	DQ522544	JN049821	DQ518758	DQ522424	DQ522327	Spatafora et al. (2007); Kepler et al. (2012b)
Pochoniachlamydosporiavar.catenulata	CBS 504.66	AF339593	AJ292398	AF339544	EF469120	EF469069	Zare et al. (2000); Sung et al. (2001); Sung et al. (2007a)
Pochoniachlamydosporiavar.catenulata	JCM 18598	AB758248	AB709837	AB709810	AB758686	AB758456	Nonaka et al. (2013)
	JCM 18600	AB758266	AB709839	AB709812	AB758704	AB758474	Nonaka et al. (2013)
Pochoniachlamydosporiavar.chlamydosporia	JCM 18605	AB758261	AB709844	AB709817	AB758699	AB758469	Nonaka et al. (2013)
	JCM 18607	AB758270	AB709846	AB709819	AB758708	AB758478	Nonaka et al. (2013)
Pochoniachlamydosporiavar.ellipsospora	JCM 18609	AB758257	AB709848	AB709821	AB758695	AB758465	Nonaka et al. (2013)
	JCM 18611	AB758265	AB709850	AB709823	AB758703	AB758473	Nonaka et al. (2013)
Pochoniachlamydosporiavar.spinulospora	JCM 18613	AB758258	AB709854	AB709827	AB758696	AB758466	Nonaka et al. (2013)
	JCM 18619	AB758272	AB709857	AB709830	AB758710	AB758480	Nonaka et al. (2013)
*Pochoniaglobispora*	CBS 203.86	–	MH861942	MH873631	–	–	Vu et al. (2019)
***Pochoniasinensis***	**ZY 22.009**	**OQ709251**	**OQ709257**	**OQ709263**	**OQ719624**	**OQ719629**	**This study**
	**ZY 22.010**	**OQ709252**	**OQ709258**	**OQ709264**	**OQ719625**	**OQ719630**	**This study**
*Pseudometarhiziumaraneogenum*	DY101741	–	MW730532	MW730618	MW753030	MW753037	Chen et al. (2022)
	DY101801	–	MW730536	MW730623	MW753032	MW753039	Chen et al. (2022)
*Pseudometarhiziumlepidopterorum*	SD05361	–	MW730543	MW730624	–	MW753041	Chen et al. (2022)
	SD05362	–	MW730611	MW730629	–	MW753042	Chen et al. (2022)
*Purpureomyceskhaoyaiensis*	BCC 1376	KX983468	–	KX983462	KX983465	KX983457	Luangsa-ard et al. (2017b)
*Purpureomycesmaesotensis*	BCC 89300	–	MN781917	MN781876	–	MN781733	Mongkolsamrit et al. (2020a)
	BCC 88441	–	MN781916	MN781877	MN781824	MN781734	Mongkolsamrit et al. (2020a)
*Purpureomycespyriformis*	BCC 85074	–	MN781929	MN781873	MN781821	MN781730	Mongkolsamrit et al. (2020a)
*Regiocrellacamerunensis*	ARSEF 7682	–	–	DQ118735	–	DQ118743	Chaverri et al. (2005b)
*Rotiferophthoraangustispora*	CBS 101437	AF339584	AJ292412	AF339535	DQ522460	AF543776	Zare et al. (2000); Sung et al. (2001); Currie et al. (2003); Spatafora et al. (2007)
*Samuelsiachalalensis*	CUP 067856	–	–	EU392637	–	EU392691	Chaverri et al. (2008)
*Samuelsiamundiveteris*	BCC 40021	–	–	GU552152	–	GU552145	Mongkolsamrit et al. (2017)
*Samuelsiarufobrunnea*	CUP 067858	–	–	AY986918	–	AY986944	Chaverri et al. (2005a)
*Shimizuomycesparadoxus*	EFCC 6279	EF469131	JN049847	EF469084	EF469117	EF469071	Sung et al. (2007ab); Kepler et al. (2012b)
	EFCC 6564	EF469130		EF469083	EF469118	EF469072	Sung et al. (2007ab)
*Sungiayongmunensis*	EFCC 2131	EF468977	JN049856	EF468833	–	EF468770	Sung et al. (2007a); Kepler et al. (2012b)
	EFCC 2135	EF468979	–	EF468834	–	EF468769	Sung et al. (2007a)
*Tyrannicordycepsfratricida*	TNS 19011	JQ257022	–	JQ257023	JQ257021	JQ257028	Kepler et al. (2012a)
*Ustilaginoideadichromenae*	MRLIB 9228	–	–	–	JQ257018	JQ257025	Kepler et al. (2012a)
*Ustilaginoideavirens*	ATCC 16180	–	–	–	JQ257019	JQ257026	Kepler et al. (2012a)
	MAFF 240421	–	JQ349068	JQ257011	JQ257017	JQ257024	Kepler et al. (2012a)
*Yosiokobayasiakusanagiensis*	TNS-F18494	JF415954	JN049873	JF415972	–	JF416014	Kepler et al. (2012a)
*Pleurocordycepsaurantiaca*	MFLUCC 17-2113	MG136904	MG136916	MG136910	MG136870	MG136875	Xiao et al. (2018)
*Pleurocordycepsmarginaliradians*	MFLU 17-1582	MG136908	MG136920	MG136914	MG271931	MG136878	Xiao et al. (2018)

Sequences highlighted in bold were generated in this study.

### ﻿Phylogenetic analyses

Lasergene software (version 6.0, DNASTAR) was used to analyse the ambiguous bases of the PCR amplicon sequences. The SSU, ITS, LSU, *RPB2* and *EF1A* sequences were retrieved from the GenBank database, based on previous studies by Mongkolsamrit et al. (2018, 2020b, 2021), Gao et al. (2021), Chen et al. (2022) and others (Table 2). The sequences for individual loci were aligned using the MAFFT multiple sequence alignment software version 7.037b (Katoh and Standley 2013) and modified manually using the MEGA software version 6.06 (Tamura et al. 2013). The SSU, ITS, LSU, *RPB2* and *EF1A* sequences were then combined using the “Concatenate Sequence” function in the PhyloSuite version 1.2.3 (Xiang et al. 2023). The best-fit substitution model was selected for the Bayesian analysis and the Maximum Likelihood analysis using the corrected Akaike Information Criterion (AICc) in the ModelFinder (Kalyaanamoorthy et al. 2017).

In the present study, the combined loci were analysed using the Bayesian Inference (BI) and the Maximum Likelihood (ML) methods. MrBayes version 3.2 (Ronquist et al. 2012) was used for the BI analysis. The Markov Chain Monte Carlo (MCMC) method was used to perform 10^8^ simulations with a sampling frequency of 10^3^ generations and a 25% burn-in. ML analysis was performed using the IQ-TREE software version 1.6.11 (Nguyen et al. 2015) and 10^4^ bootstrap (BS) tests were performed using the ultrafast algorithm (Minh et al. 2013). The BI and ML analyses were performed in the PhyloSuite platform version 1.2.3 (Xiang et al. 2023).

## ﻿Results

### ﻿Phylogenetic analyses

*Pleurocordycepsaurantiacus* (MFLUCC 17-2113) and *P.marginaliradians* (MFLU 17-1582) were used as the outgroup for the phylogenetic analysis. The concatenated sequences (SSU, ITS, LSU, *RPB2* and *EF1A*) included 113 taxa and consisted of 3,368 nucleotides (SSU, 905 bp; ITS, 448 bp; LSU, 453 bp; *RPB2*, 756 bp; and *EF1A*, 806 bp) with inserted gaps (Suppl. material 1). ModelFinder was used to obtain the best-fit substitution model, based on the AICc algorithm and are listed in Suppl. material 2.

**Figure 1.**
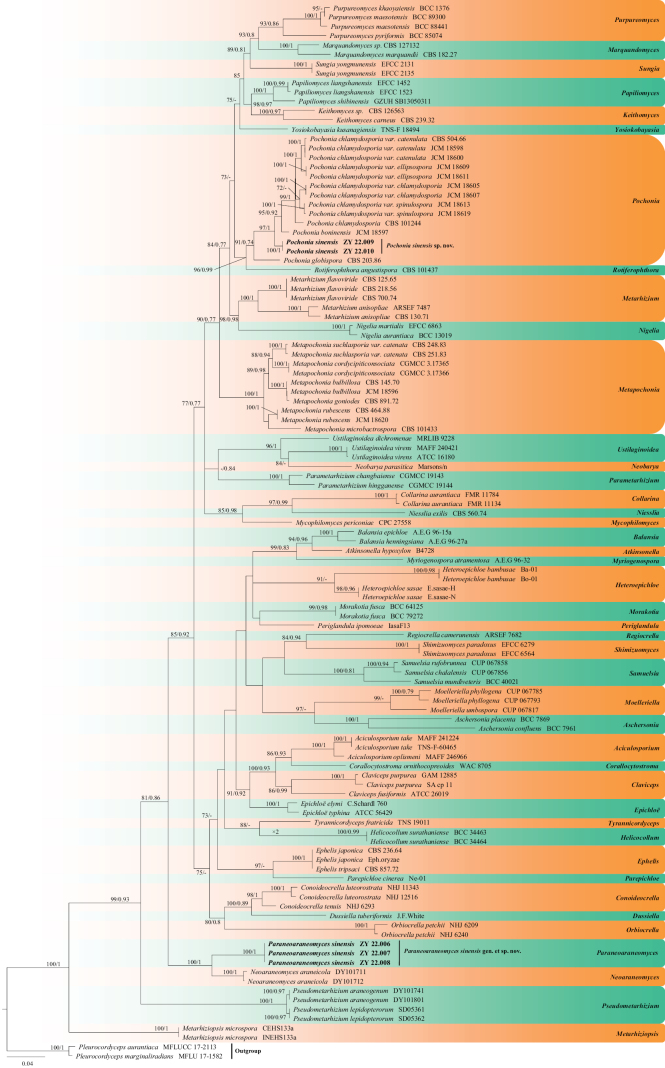
Phylogram based on the Maximum Likelihood (ML) analysis using the SSU, ITS, LSU, *RPB2* and *EF1A* sequences of Clavicipitaceae. The ML bootstrap values (≥ 70%) and the Bayesian posterior probability values (≥ 0.70) are indicated along the branches (BP/ML). The new taxa are highlighted in bold.

The phylogenetic trees (Fig. 1) constructed according to the ML and BI analyses were largely congruent and strongly supported in most clades. Most genera were clustered into independent clades (Chen et al. 2022; Fig. 1). Two new isolates, ZY 22.009 and ZY 22.010, belonged to a new species below named *Pochoniasinensis*. They were clustered into a single clade with high support value (100% BS support [BS]/1 posterior probability [PP]) under the genus *Pochonia*. The genus *Pochonia* was closely related to *Rotiferophthora* (Fig. 1). This result was in agreement with the previous studies by Kepler et al. (2014) and Chen et al. (2022). Furthermore, the remaining three new isolates, ZY 22.006, ZY 22.007 and ZY 22.008 clustered into another independent clade with a high support value (100% BS/1 PP) and showed a close relationship with *Neoaraneomyces*.

## ﻿Taxonomy

### ﻿ Paraneoaraneomyces

Taxon classificationFungiHypocrealesClavicipitaceae

Zhi.Y. Zhang & Y.F. Han gen. nov.

1D693499-AEEA-5F0B-8D9C-0D1151C9E46E

848089

#### Etymology.

Based on its close phylogenetic relationship to *Neoaraneomyces*.

#### Geographical distribution.

China.

#### Description.

Saprobic in soil. ***Sexual morph***: not observed. ***Asexual morph***: *Hyphae* hyaline, smooth, branched, septate. *Phialides* arising from aerial hyphae or hyphae regimental, solitary, straight to flexuous, tapering with enlarged base, smooth, hyaline. *Conidia* borne on the apex of the phialides or in small globose heads at the apices of the phialides. *Conidia* cymbiform to reniform, smooth-walled, one-celled, adhering in globose heads or the apex of phialides.

#### Type species.

*Paraneoaraneomycessinensis* Zhi. Y. Zhang & Y. F. Han.

#### Notes.

Currently, the family Clavicipitaceae includes 52 genera and more than 500 species (Hyde et al. 2020; Mongkolsamrit et al. 2020a, 2021; Gao et al. 2021; Chen et al. 2022). Of these genera, no SSU, ITS, LSU, *RPB2* and *EF1A* sequences are available for the genera *Cavimalum*, *Epicrea*, *Helminthascus*, *Konradia*, *Loculistroma*, *Mycomalus* and *Neocordyceps*, while the sequences for the genera *Nigrocornus*, *Pseudomeria* and *Romanoa* are unverified or lacking (https://www.ncbi.nlm.nih.gov/, accessed on 8 May 2023). Therefore, we could not compare the phylogenetic relationships between these genera and *Paraneoaraneomyces*. In addition, amongst these genera, *Cavimalum*, *Epicrea*, *Helminthascus*, *Konradia*, *Mycomalus* and *Sphaerocordyceps* no asexual morph has been reported (White et al. 2003; Hyde et al. 2020). We, therefore, have not been able to compare the morphological characteristics between these genera and *Paraneoaraneomyces*. Phylogenetically, *Paraneoaraneomycessinensis* represents a well-supported monophyletic lineage in the family Clavicipitaceae and closely related to *Neoaraneomyces* (Fig. 1). Morphologically, *Paraneoaraneomyces* can be distinguished from other genera in the family Clavicipitaceae by the cymbiform to reniform conidia adhering to the apex of the phialides or in the form of small globose heads at the apex of the phialides and the phialides were solitary, straight to flexuous and arose from the aerial or regimental hyphae.

### ﻿ Paraneoaraneomyces sinensis

Taxon classificationFungiHypocrealesClavicipitaceae

Zhi. Y. Zhang & Y. F. Han, sp. nov.

4618CF8E-7A49-5523-99E6-0DF326FF2C4A

848160

Fig. 2

#### Etymology.

After the country of origin.

#### Type.

Kaili City, Guizhou Province, China; 27°17’56’’N, 108°82’68’’E; isolated from the green belt soil in July 2022; Zhi-Yuan Zhang (holotype ZY H-22.006, ex-holotype ZY 22.006, *ibid.*, ZY 22.007).

#### Geographical distribution.

Guizhou Province, China.

#### Description.

Culture characteristics (14 days at 25 °C): ***Colonies*** on PDA 35–37 mm in diameter, white, slightly raised at centre, fluffy, nearly round, margin regular; reverse: pale yellow. ***Colonies*** on MEA 35–37 mm in diameter, white, plicated, flocculent, nearly round, margin regular; reverse: pale yellow. ***Colonies*** on SNA 29–31 mm in diameter, white, flat, felty, nearly round, margin regular; reverse: white, compact at centre. ***Colonies*** on OA 36–38 mm in diameter, white, felty, early round, margin regular; reverse: white.

**Figure 2.**
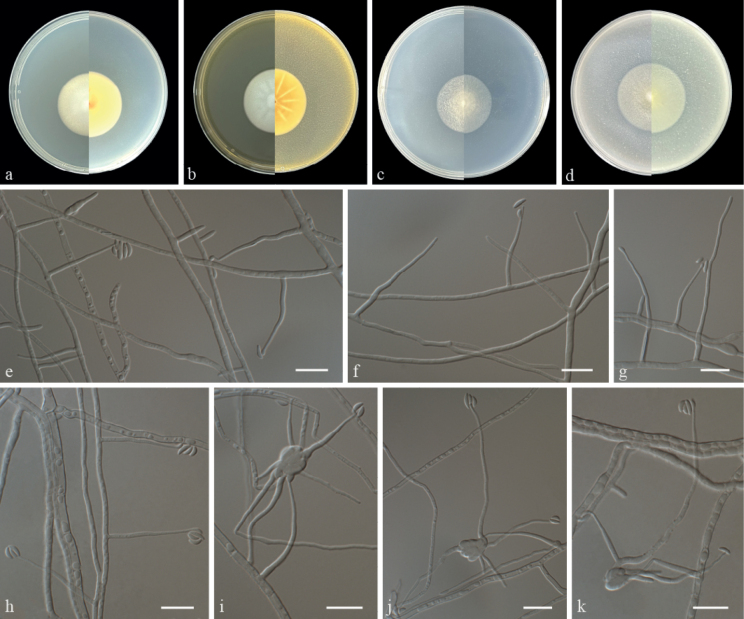
Morphology of *Paraneoaraneomycessinensis* sp. nov. **a–d** colony on PDA, MEA, SNA and OA after 14 d at 25 °C (upper surface and lower surface) **e–h** phialides, conidia **i–k** phialides are arising from hyphae regimental, conidia. Scale bars: 10 μm (**e–k**).

***Hyphae*** hyaline, smooth, branched, septate, 1.0–3.0 μm in diameter. ***Phialides*** arising from aerial hyphae or hyphae regimental, solitary, straight to flexuous, tapering with enlarged base, smooth, hyaline, 19.0–34.0 × 0.5–1.5 µm (av. 27.0 × 1.1, n = 50). ***Conidia*** borne on the apices of the phialides or in small globose heads at the apex of the phialides. ***Conidia*** cymbiform to reniform, smooth-walled, one-celled, adhering in globose heads or the apex of phialides, 3.0–5.5 × 1.0–1.5 µm (av. 4.3 × 1.4, n = 50). ***Sexual morph*** undetermined.

#### Additional material examined.

Kaili City, Guizhou Province, China; 27°17’72’’N, 108°83’10’’E; isolated from the green belt soil in July 2022; Zhi-Yuan Zhang, ZY 22.008.

#### Notes.

The multi-locus phylogenetic analyses showed that *Paraneoaraneomycessinensis* is closely related to *Neoaraneomycesaraneicola* (Fig. 1), but can be distinguished, based on differences in their sequence similarity. The ITS sequence of *P.sinensis* showed 93.6% similarity, differences in 13 base pairs (bp) and 22 gaps when compared to the 551 bp ITS sequence of *N.araneicola*DY101711 (Type strain). The LSU sequence of *P.sinensis* showed 99.3% similarity, differences in 5 bp and without gaps when compared to the 832 bp LSU sequence of *N.araneicola*DY101711. The *RPB2* sequence of *P.sinensis* showed 83.9% similarity, differences in 158 bp and 8 gaps when compared to the 1,034 bp *RPB2* sequence of *N.araneicola*DY101711. The *EF1A* sequence of *P.sinensis* showed 96.2% similarity, differences in 35 bp and without gaps when compared to the 937 bp *EF1A* sequence of *N.araneicola*DY101711. Morphologically, the phialides of *P.sinensis* were solitary, straight to flexuous, arising from the aerial or regimental hyphae compared to the phialides of *N.araneicola* that were solitary or in groups of two to four and arose from the aerial hyphae (Chen et al. 2022). Furthermore, the conidia of *P.sinensis* were cymbiform to reniform and adhering to the apex of the phialides or in small globose heads at the apex of the phialides compared with fusiform to ellipsoidal conidia that were arranged as chains in *N.araneicola* (Chen et al. 2022).

### ﻿ Pochonia sinensis

Taxon classificationFungiHypocrealesClavicipitaceae

Zhi. Y. Zhang & Y. F. Han, sp. nov.

EF90F62C-FFB5-55BF-AED4-302CA95AFBB6

848088

Fig. 3

#### Etymology.

After the country of origin.

#### Type.

Kaili City, Guizhou Province, China; 27°17’56’’N, 108°82’68’’E; isolated from the green belt soil in July 2022; Zhi-Yuan Zhang (holotype ZY H-22.009, ex-holotype ZY 22.009, *ibid.*, ZY 22.010).

#### Geographical distribution.

Guizhou Province, China.

#### Description.

Culture characteristics (14 days at 25 °C): ***Colonies*** on PDA fast-growing, reaching 74–77 mm in diameter, white, flat, fluffy to flocculent, margin identiﬁed; reverse: white. ***Colonies*** on MEA 67 mm in diameter, white, flat, compact, fluffy to flocculent, margin identiﬁed; reverse: white. ***Colonies*** on SNA 59–60 mm in diameter, white, aerial mycelia sparse, flat, flocculent, nearly round, margin regular; reverse: white. ***Colonies*** on OA 58 mm in diameter, white, aerial mycelia sparse, flat, felty, nearly round; reverse: white.

**Figure 3.**
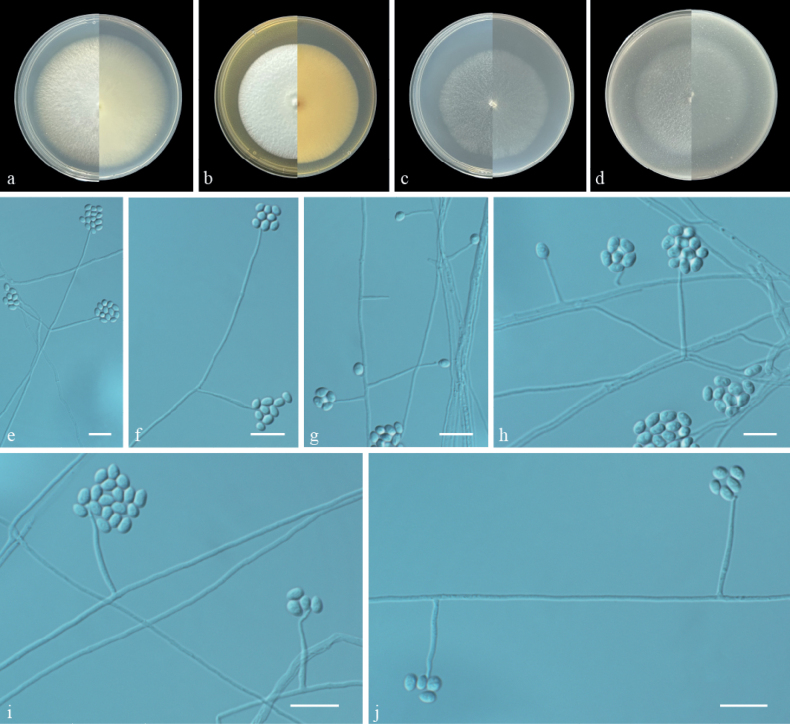
Morphology of *Pochoniasinensis* sp. nov. **a–d** colony on PDA, MEA, SNA and OA after 14 d at 25 °C (upper surface and lower surface) **e–j** phialides, conidia. Scale bars: 10 μm (**e–j**).

***Hyphae*** hyaline, smooth, branched, septate, 0.5–1.5 μm in diameter. ***Phialides*** produced from prostrate aerial hyphae, solitary or rarely in whorls of 2–3, slender, tapering towards the tip, 5.5–51.0 × 0.5–1.5 µm (av. 22.0 × 1.0, n = 50). ***Conidia*** in small globose heads at the apex of the phialides. ***Conidia*** ovoid, sometimes subglobose or ellipsoidal, smooth-walled, one-celled, adhering in globose heads, 3.0–4.5 × 2.0–3.0 µm (av. 3.6 × 2.5, n = 50). Swollen hyphae not observed. ***Dictyochlamydospores*** not observed. ***Crystals*** absent. ***Sexual morph*** undetermined.

#### Notes.

The multi-locus phylogenetic analyses (Fig. 1) and morphological characteristics showed that ZY 22.009 and ZY 22.010 represent a new species of *Pochonia*. Morphologically, *P.sinensis* shared similar morphological characters with *P.globispora* and *P.boninensis*, but does not produce dictyochlamydospores (Zare and Gams 2007; Nonaka et al. 2013). However, *P.sinensis* can be easy distinguished from *P.globispora* and *P.boninensis*, based on the ovoid conidia and the absence of irregularly swollen hyphae (Zare and Gams 2007; Nonaka et al. 2013).

## ﻿Discussion

In this study, we proposed a new *Pochonia* species and a new genus *Paraneoaraneomyces* within the family Clavicipitaceae. This study has important implications for the species diversity, taxonomy and geographic distribution of Clavicipitaceae (Hypocreales).

Fungi are highly abundant eukaryotes (Purvis and Hector 2000) with significant diversity and cosmopolitan distribution and play an essential role in the functions and processes of a wide variety of ecosystems. However, only 150,000 fungal species have been described to date and its plausible that several fungal genera and species are yet to be discovered. Taxonomy is a fundamental discipline of naming, describing and classifying a living organism, plant or fungus and represents the initial step towards understanding its biodiversity, ecological niche and biotechnological utility (Yasanthika et al. 2022). An increasing number of new fungal taxa are constantly being discovered, but mycotaxonomy of a new fungal species is challenging (Aime et al. 2021). Currently, integration of multiple methods is recommended for the taxonomic classification of newly-identified fungal species. Amongst these methods, morphological characteristics and phylogenetic analysis are of primary importance in addition to the ecological habitats, as well as the physiological and biochemical characteristics. The family Clavicipitaceae includes many entomopathogenic fungi, but only a small number of taxa are parasitic and most others show diverse nutritional patterns. Therefore, utmost care is necessary when classifying a new fungal isolate, based on the substrate or a parasitic fungus on an insect host. All the isolates obtained in this study were isolated from soil. Further investigations are necessary to determine if these new fungal isolates were parasitic to insects.

Soil is the largest natural reservoir of microorganisms and is inhabited by a large number of fungi. Taxonomy of soil fungi is an emerging area of research. Currently, only about 800,000 species of soil fungi have been identified worldwide (Senanayake et al. 2022). Majority of studies have focused on the diversity of fungi in the forest, silt, riparian, coastal and contaminated soils (Fracetto et al. 2013; Frac et al. 2018; Satyanarayana et al. 2019), but relatively little is known regarding the fungal taxa in the urban soils. Taxonomic studies of soil fungi use both culture-dependent and non-culture-dependent methods. The culture methods are of great interest because the isolated strains can be used to obtain genetic sequence and morphological data in applied research (Yasanthika et al. 2022). The new fungi described in this study were all isolated from soil and their ecological functions and applications are worthy of further study.

### ﻿Key to the genus *Paraneoaraneomyces* and its related genera (Revised from Maharachchikumbura et al. (2016)).

**Table:** 

1	Host is a plant	**2**
–	Host insects, nematodes, rotifers, protozoans or soil	**3**
2	Asexual morph produced	***Metarhiziopsis***
–	Sexual morph produced	**4**
3	Conidia with adhesive hapteron	***Pseudomeria***
–	Conidia without adhesive hapteron	**5**
4	Stromata stalked	***Neocordyceps***
–	Stromata lacking stalks	**6**
5	Conidia cymbiform to reniform	***Paraneoaraneomyces***
–	Conidia fusiform or ellipsoidal	**7**
6	Host bamboo	***Loculistroma***
–	Host grasses	***Nigrocornus***
7	Conidiophores mononematous	***Neoaraneomyces***
–	Conidiophores synnematous or mononematous	***Pseudometarhizium***

## Supplementary Material

XML Treatment for Paraneoaraneomyces

XML Treatment for Paraneoaraneomyces sinensis

XML Treatment for Pochonia sinensis

## ﻿Additional information

### Author ORCIDs

Zhi-Yuan Zhang https://orcid.org/0000-0003-2031-7518

Yao Feng https://orcid.org/0000-0002-0888-8775

Shuo-Qiu Tong https://orcid.org/0009-0006-9422-5551

Chen-Yu Ding https://orcid.org/0000-0003-2639-3300

Gang Tao https://orcid.org/0000-0002-0882-3752

Yan-Feng Han https://orcid.org/0000-0002-8646-3975

### Data availability

All of the data that support the findings of this study are available in the main text or Supplementary Information.

## Supplementary materials

Supplementary material 1 Sequence dataset Data type sequence https://binary.pensoft.net/file/860999This dataset is made available under the Open Database License (http://opendatacommons.org/licenses/odbl/1.0/). The Open Database License (ODbL) is a license agreement intended to allow users to freely share, modify, and use this Dataset while maintaining this same freedom for others, provided that the original source and author(s) are credited.Zhi-Yuan Zhang, Yao Feng, Shuo-Qiu Tong, Chen-Yu Ding, Gang Tao, Yan-Feng Han

Supplementary material 2 The best-fit evolutionary model in the phylogenetic analyses Data type table https://binary.pensoft.net/file/861000This dataset is made available under the Open Database License (http://opendatacommons.org/licenses/odbl/1.0/). The Open Database License (ODbL) is a license agreement intended to allow users to freely share, modify, and use this Dataset while maintaining this same freedom for others, provided that the original source and author(s) are credited.Zhi-Yuan Zhang, Yao Feng, Shuo-Qiu Tong, Chen-Yu Ding, Gang Tao, Yan-Feng Han
