# Supplementary material for: ﻿Morphological and phylogenetic characterisation of two new soil-borne fungal taxa belonging to Clavicipitaceae (Hypocreales, Ascomycota)
Source: MycoKeys. 2023 Jun 7;98:113–32. doi: 10.3897/mycokeys.98.106240 (PMC10267719; doi:10.3897/mycokeys.98.106240)
Supplement: Supplementary material 2 — The best-fit evolutionary model in the phylogenetic analyses [file mycokeys-98-113-s002.docx]

**Table S1.** The best-fit evolutionary model in the phylogenetic analyses.

| **Phylogenetic analysis** | **Model** | | | | | |
| --- | --- | --- | --- | --- | --- | --- |
|  | **SSU** | **ITS** | **LSU** | | ***RPB2*** | ***EF1A*** |
| ML analysis | K2P+I+I+R2 | GTR+F+I+I+R4 | TIM2+F+I+I+R3 | TIM3+F+I+I+R4 | | TIM3+F+I+I+R4 |
| BI analysis | K2P+I+G4 | GTR+F+I+G4 | GTR+F+I+G4 | GTR+F+I+G4 | | GTR+F+I+G4 |

ML: Maximum likelihood; BI: Bayesian inference.
